# Determinants of Patient Satisfaction with Vascular Access in Hemodialysis: Insights from a Multicenter Study in Italy

**DOI:** 10.3390/clinpract15110203

**Published:** 2025-10-31

**Authors:** Vincenzo Andretta, Marco Cascella, Alexia Cerrone, Angela Prendin, Antonio Mastrangelo, Valentina Cerrone

**Affiliations:** 1 Department of Medicine, Surgery and Densistry, University of Salerno, 84084 Baronissi, Italy; mcascella@unisa.it (M.C.); a.cerrone19@studenti.unisa.it (A.C.); 2 Palliative Care and Antalgic Therapy/Pediatric Hospice, University Hospital of Padua, 35122 Padua, Italy; angela.prendin@aopd.veneto.it; 3 Dialysis Unit, Luigi Curto Hospital, 84035 Polla, Italy; mastrangelo.ant@tiscali.it

**Keywords:** hemodialysis, patient-reported outcomes, SF-VAQ, quality of life

## Abstract

**Background:** Vascular access is a very important element for patients on chronic hemodialysis treatment, but it is also a major source of complications, often compromising patients’ quality of life. Arteriovenous fistulas (AVFs) are preferred for their durability, but complications such as edema, bruising, cannulation pain, and hygiene concerns can affect patient satisfaction. **Aim**: We aimed to evaluate patient satisfaction with vascular access and to identify the clinical and sociodemographic factors influencing this satisfaction. **Methods:** We conducted a multicenter cross-sectional study on 235 hemodialysis patients in Italy. Satisfaction was assessed using the Short Form Vascular Access Questionnaire (SF-VAQ). Clinical and sociodemographic data were collected and analyzed with descriptive statistics, correlations, and multivariate regression models. **Results:** Satisfaction was significantly influenced by local complications, perceived hygiene, and access duration. Lower satisfaction was reported by patients with swelling, bruising, or negative hygiene perceptions. Longer use of the access was also associated with declining satisfaction. **Conclusions:** Patient satisfaction involves both clinical outcomes and patient perceptions. The integration of patient-reported outcomes (PROs) into vascular access management can help clinicians identify early dissatisfaction and implement interventions that can improve treatment adherence and quality of life.

## 1. Introduction

Vascular access is essential for patients undergoing hemodialysis and represents a key determinant of treatment adequacy and quality of life [1]. The longevity and hemodialysis effectiveness depend on the quality and functionality of vascular access, which directly influences the adequacy of the treatment and patient outcomes. However, vascular access is also a major source of complications, contributing to increased morbidity, hospitalization rates, and healthcare costs [2].

Maintaining vascular access patency to ensure effective dialysis remains a challenge across all types. The complications are substantially different between catheters and fistulas [3,4], but these differences were not specifically addressed in this study. The native arteriovenous fistula (AVF) is generally considered the optimal access because it has lower infection and thrombosis rates and superior long-term patency compared to arteriovenous grafts (AVGs) and central venous catheters (CVCs) [5,6,7]. Nonetheless, AVGs and CVCs remain necessary in cases of vascular failure, inadequate anatomy, or urgent dialysis initiation.

Patient-reported outcomes (PROs) have become increasingly important in evaluating healthcare quality [8,9]. In this context, patient satisfaction with vascular access should be distinguished from specific perceptions such as pain, edema, hygiene, and impact on daily living [10]. Satisfaction is a higher-order construct encompassing not only symptoms, but also expectations, trust in care, and perceived quality, as highlighted in frameworks such as ICHOM. Negative experiences may lead to frustration, treatment fatigue, and reduced adherence to dialysis regimens [11,12].

Although the relationship between vascular access complications and patient satisfaction has been described, its broader clinical implications remain underexplored. Common complications such as edema, bleeding, bruising, cannulation failures, and hospitalizations are consistently linked to lower satisfaction [13,14]. Some studies suggest patients adapt over time, while others describe increasing frustration with prolonged access use [15].

Therefore, the aim of this study is to explore patient satisfaction with vascular access in a large Italian cohort, integrating clinical indicators with PROs to identify determinants of satisfaction. By combining clinical, psychosocial, and functional dimensions, the study seeks to generate evidence that can inform care optimization, minimize burden, and enhance the dialysis experience.

This work contributes to the literature as one of the few to assess vascular access satisfaction in the Italian hemodialysis population. The inclusion of detailed sociodemographic variables and the use of a patient-centered Likert-scale questionnaire provide a novel multidimensional perspective on vascular access satisfaction, addressing an area still underrepresented in European research.

## 2. Materials and Methods

### 2.1. Study Design and Objectives

This multicenter, cross-sectional observational study was conducted in accordance with the STROBE (Strengthening the Reporting of Observational Studies in Epidemiology) guidelines (Supplementary Table S1). The research was carried out over a six-month period (September 2024–February 2025) across three dialysis centers located in southern Italy (Campania, Puglia, and Basilicata). Data were collected during routine dialysis sessions to ensure minimal disruption to patients’ clinical routines and to preserve ecological validity.

The primary aim of the study was to evaluate patient satisfaction with vascular access through a patient-reported outcome (PRO) approach. To this end, we employed the Short Form-Vascular Access Questionnaire (SF-VAQ), a concise and validated instrument specifically designed to measure patients’ satisfaction and perceived symptoms related to vascular access. Its brevity, clarity, and strong psychometric properties make it particularly suitable for clinical populations undergoing regular hemodialysis.

The specific objectives were as follows:

1. To describe patient-reported satisfaction with vascular access.
2. To examine the association between perceived vascular access complications—such as pain, edema, bruising, and bleeding—and overall satisfaction.

### 2.2. Participants, Inclusion Criteria and Exclusion Criteria

Eligible participants were adults aged 18 years or older who had been receiving hemodialysis for at least 12 months and were able to comprehend and complete the questionnaire. Participation was voluntary. Information on the type of vascular access (arteriovenous fistula vs. central venous catheter) was collected; however, due to limitations in sample size, the present analysis did not stratify results by access type.

Eligible patients were identified during scheduled dialysis sessions by nephrologists and dialysis nurses. Based on the inclusion and exclusion criteria, the patient was contacted during routine treatment and informed about the study. We obtained written informed consent before completing the questionnaire, which was administered in paper format during dialysis sessions.

While variables such as marital status and number of children may appear ancillary, they provide valuable insights into social support networks that could influence patients’ perceptions of vascular access care and overall satisfaction.

Support from healthcare professionals was available for participants requiring assistance in completing the survey. None of the patients invited to participate reported cognitive or linguistic impairments that might have hindered their understanding of the questionnaire. All participants received detailed information about the study’s objectives and procedures. Written informed consent was obtained, and participants were informed of their right to withdraw from the study at any time without any impact on their ongoing treatment.

Although data on comorbidities were collected, specific information on body mass index (BMI) and diabetes status was not included. This constitutes a limitation, as both conditions are known to affect vascular access cannulation and may influence patient satisfaction.

Patients with cognitive impairment, language barriers preventing comprehension of the questionnaire, and refusal to provide informed consent were excluded.

The target sample size was approximately 230–250 patients, based on the expected eligible population in the three participating centers during the recruitment period. We did not perform a formal sample size calculation, as the study had an exploratory, descriptive design.

### 2.3. Data Collection and Measurement Instrument

Data collection was conducted using a structured questionnaire. The tool was pre-tested to enhance its validity and reliability, and modifications were made accordingly based on pre-test results. The questionnaire was designed around the dependent variables—namely, the factors influencing patient satisfaction with vascular access.

For each participant, the type of vascular access was recorded. Both arteriovenous fistulas (AVFs) and central venous catheters (CVCs) were included in the cohort. However, subgroup analyses based on access type were not performed due to limitations in sample size.

The SF-VAQ, a validated instrument, was used to assess patient satisfaction. Since the full version is already published and cross-culturally validated, it was not annexed to this manuscript but is available in the original validation study [16]. The SF-VAQ allows a structured assessment of symptoms, functional limitations, and overall satisfaction, providing insights beyond clinical indicators. Each item in the SF-VAQ is rated on a 7-point Likert scale ranging from 1 (“not satisfied at all” or “severely affected”) to 7 (“completely satisfied” or “not affected at all”), depending on the item. General satisfaction was measured using item Q1, which directly assesses overall satisfaction with the vascular access. Items Q2 to Q13 addressed specific dimensions, including pain, bleeding, edema, bruising, hygiene, activities of daily living (ADLs), rest/sleep, and self-image. For analytic purposes, responses of 6 or 7 on Q1 were classified as “high satisfaction” (or “high agreement”), in accordance with previous literature; scores ≤5 were considered indicative of moderate to low satisfaction.

The item referring to “rest” assessed the patient’s ability to sleep and rest comfortably despite the presence of vascular access. Hygiene perception was evaluated through a specific item that asked whether the access site appeared clean—free from redness, exudate, unpleasant odor, or visible dirt. This item did not assess clinical infection, but rather the patient’s subjective impression of cleanliness. The SF-VAQ was selected based on a structured literature review conducted with the assistance of a medical librarian.

Comorbidities were self-reported and subsequently verified through medical records.

To minimize selection and measurement bias, all eligible patients were invited to participate. Questionnaires were administered by trained dialysis nurses following standardized procedures. Patients completed the questionnaires on paper, and responses were transcribed into an electronic database by an independent nurse (V.A.). During data transcription, minor discrepancies such as illegible handwriting or inconsistent dates were flagged and resolved by consensus within the research team to ensure accuracy and integrity. No changes were made to patients’ original responses. The datasets used and/or analyzed during the current study are available from the corresponding author upon reasonable request.

Socio-demographic variables were also collected to assess whether individual or family-related characteristics influenced patients’ perceptions of vascular access and overall satisfaction.

We assigned a unique alphanumeric code to each questionnaire. The same code was used to extract relevant clinical data from medical records to establish the link between patient-reported outcomes and clinical variables. After data integration, identifying information was permanently removed, and we utilized only anonymized dataset for statistical analysis.

This study was designed to focus exclusively on patient-reported outcomes (PROs). For this reason, objective clinical and laboratory variables such as causes of chronic kidney disease, dialysis adequacy parameters (e.g., Kt/V), and history of previous vascular accesses or transplantations were not collected. The rationale was to isolate the patient-reported experience, measured through the SF-VAQ, from clinical endpoints that have been extensively addressed in previous research.

### 2.4. Variables

The study variables were grouped into three main categories: (a) sociodemographic (age, gender, marital status, education, employment, living situation, caregiver availability); (b) clinical (dialysis duration, vascular access type, comorbidities, number of children); and (c) patient-reported outcomes from the SF-VAQ (general satisfaction, pain, bleeding, edema, bruising, hygiene, ADLs, appearance, rest, problems, care, hospitalization, and vascular access duration).

### 2.5. Data Management and Quality Control

Following data transcription, a double-check procedure was conducted to identify any potential inconsistencies or missing values. No missing data were identified, as all participants had fully completed the administered questionnaires. Information regarding comorbidities was collected through medical record review and confirmed by clinical staff. The relatively high proportion of patients without comorbidities may reflect potential underreporting or selection bias.

As no missing values were detected, no imputation procedures were required. All statistical analyses were conducted using complete case datasets. In compliance with ethical standards, all data were anonymized and securely stored, with access limited to authorized study personnel. Data protection procedures adhered strictly to the European General Data Protection Regulation (GDPR, EU Regulation 2016/679) [17].

### 2.6. Statistical Analysis

Statistical analyses were performed by a biostatistics expert using Stata SE version 18 (StataCorp LLC, College Station, TX, USA). Continuous variables were summarized using means and standard deviations (SD), or medians and interquartile ranges (IQR), depending on their distribution. Skewness and kurtosis were also evaluated to assess the normality of data distribution. Categorical variables were described using absolute frequencies and percentages.

The relationships between variables were explored using Pearson’s correlation for normally distributed continuous data, and Spearman’s rank correlation for ordinal or non-normally distributed variables. A *p*-value < 0.05 was considered statistically significant.

Multiple linear regression models were employed to identify factors associated with patient satisfaction, adjusting for relevant covariates. Covariates were selected based on clinical relevance reported in the literature, statistical significance in bivariate analyses (*p* < 0.10), and theoretical plausibility regarding vascular access satisfaction. Variables lacking clinical or statistical relevance were excluded to maintain model parsimony. Additionally, binary logistic regression models were used to estimate the probability of high satisfaction, defined as a score of ≥6 on item Q1 of the SF-VAQ.

Model diagnostics included checks for multicollinearity, residual normality for linear regression, and the Hosmer–Lemeshow goodness-of-fit test for logistic regression models. All results were reported with 95% confidence intervals (CI), and significance was set at *p* < 0.05.

### 2.7. Ethical Considerations

This study was conducted in accordance with the principles of the Declaration of Helsinki. As data collection was observational, anonymous and non-interventional, we did not perform additional procedures beyond routine, and Italian regulation did not require formal approval from the ethics committee. However, all patients received written information and provided signed informed consent before their study participation. The study protocol was pre-registered on the Open Science Framework (OSF) and is accessible at https://doi.org/10.17605/OSF.IO/QP2WM.

## 3. Results

### 3.1. Patient Characteristics

A total of 250 patients were invited to participate. Of these, 15 were excluded because 7 refused to provide consent, 5 had less than 12 months of dialysis treatment, and 3 had cognitive or language limitations. Therefore, 235 patients provided complete responses and were included in the final analysis. Demographic and clinical characteristics are presented in Table 1.

The mean age of participants was 68.27 years (SD = 12.55), with a median age of 70 and a range from 20 to 91 years. The age distribution was slightly left-skewed (skewness = −1.07), suggesting a higher concentration of older adults. A kurtosis value of 4.79 indicated a leptokurtic distribution with more extreme age values than expected under normal distribution.

The average dialysis duration was 6.05 years (SD = 5.70), with a median of 4 years and a range of 1 to 36 years. The distribution was positively skewed (skewness = 2.30), indicating that most patients had relatively recent vascular access, while a smaller proportion had long-term access. The average number of children was 1.78 (SD = 1.17), with a median of 2 and a maximum of 11. This variable also showed high skewness (2.05) and kurtosis (18.40).

The sample was predominantly male (60.85%) and married (76.17%). Most patients had children (86.38%) and lived with a spouse and children (40.85%). The most frequent vascular access type was a native AVF (61.28%). Nearly half had no comorbidities (48.09%), and the majority (75.74%) lacked caregiver support.

Regarding the primary endpoint, overall satisfaction with vascular access (SF-VAQ Q1) was assessed. The mean score was 6.28 (SD = 1.06), with most patients reporting high satisfaction (scores ≥ 6). Domain-specific items revealed moderate impact for pain (mean = 2.32, SD = 1.81), bleeding (mean = 2.25, SD = 1.72), edema (mean = 2.24, SD = 1.78), and bruising (mean = 2.28, SD = 1.84). Hygiene perception (mean = 2.17, SD = 1.67) and ADL limitations (mean = 2.37, SD = 1.74) were also frequently reported. The lowest score was observed in the rest/sleep domain (mean = 2.13, SD = 1.55), suggesting frequent interference of vascular access with sleep quality. A complete distribution of responses for all 13 SF-VAQ items is reported in Table 2.

### 3.2. Correlation Analysis

We performed a series of correlation analyses to assess potential associations between demographic, clinical, and satisfaction-related variables (Table 3).

Age was weakly positively correlated with dialysis duration (r = 0.129, *p* < 0.05) and moderately with number of children (r = 0.352, *p* < 0.05), aligning with expected demographic patterns. Discomfort indicators—including pain, edema, and bruising—showed significant negative correlations with satisfaction, indicating that increased discomfort was associated with lower perception scores. Similarly, perception of poor hygiene and longer vascular access duration negatively correlated with satisfaction levels. These findings underline the cumulative burden of vascular access-related issues over time.

### 3.3. Multiple Linear Regression Analysis

A multiple linear regression analysis was conducted to identify the predictors of patient satisfaction with vascular access (Table 4).

Edema (β = −0.29, *p* = 0.006), bruising (β = −0.24, *p* = 0.023), poor hygiene perception (β = −0.18, *p* = 0.001), and vascular access duration (β = −0.32, *p* = 0.006) were statistically significant predictors of lower satisfaction. These findings suggest that both physical and perceptual factors substantially influence patients’ subjective evaluations of vascular access. Other variables such as age, number of children, and pain were not statistically significant in this model.

### 3.4. Logistic Regression Analysis for High Satisfaction

We conducted a binary logistic regression to explore determinants of high satisfaction (SF-VAQ Q1 ≥ 6) (Table 5).

Patients reporting poor hygiene perception had 44% lower odds of high satisfaction (OR = 0.56, *p* = 0.002). Vascular access duration was also a strong inverse predictor (OR = 0.31, *p* < 0.001), highlighting a possible deterioration in perception over time. Number of children was weakly associated with satisfaction (OR = 0.65, *p* = 0.029), though clinical implications remain unclear. No subgroup analysis was performed due to limited sample sizes in some access types.

Multicollinearity was assessed in the multiple linear regression model using the Variance Inflation Factor (VIF), which yielded a mean value of 5.26. Although edema and bruising showed VIF values above 10, they were retained due to their clinical relevance. No additional multicollinearity diagnostics were performed for the logistic regression model, but the consistency of included predictors with those of the linear model supports the robustness of the associations.

### 3.5. Predicted Probability of High Vascular Access Satisfaction by Duration of Access

**Figure 1:**
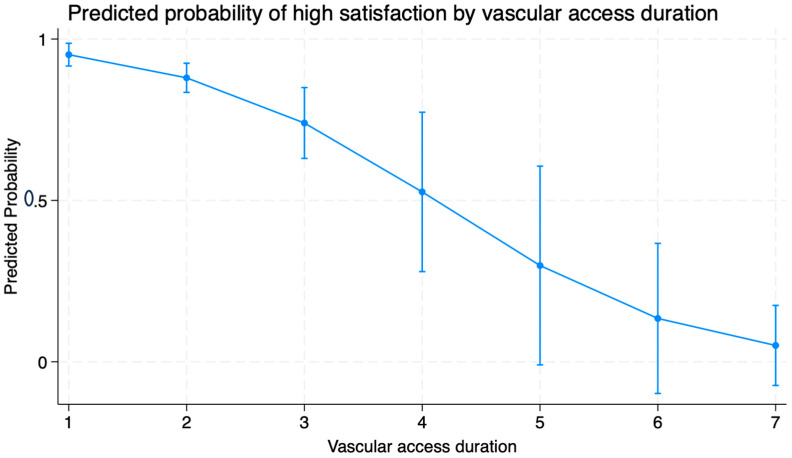
Predicted probability of high satisfaction by vascular access duration (in years). Error bars indicate 95% confidence intervals. The *X*-axis is labeled as “vascular access duration (in years)”. The *Y*-axis represents the “predicted probability” ranging from 0 to 1.

A predictive probability model was constructed to illustrate the association between vascular access duration and the likelihood of high satisfaction (Figure 1).

Patients with a 1-year access duration had a 95% predicted probability of reporting high satisfaction. In contrast, those with access in place for over 7 years had only a 5% predicted probability. As shown in Figure 1, satisfaction declines steeply with access duration, reinforcing the need for periodic reassessment. These findings suggest a steep decline in satisfaction with increasing access longevity, reinforcing the need for periodic reassessment and early intervention.

## 4. Discussion

This multicenter study provides an original contribution to nephrology by exploring patient satisfaction with vascular access in hemodialysis with the integration of clinical indicators and patient-reported outcomes (PROs). Unlike most previous studies, which focused mainly on objective clinical parameters such as patency, thrombosis and infections, our study focused on the patient experience, highlighting how satisfaction is substantially influenced by subjective factors such as perception of hygiene, pain, local complications and esthetic impact.

Vascular access guidelines recommend native arteriovenous fistulas (AVFs) as the first choice due to better patency and lower infection risks compared to arteriovenous grafts (AVGs) and central venous catheters (CVCs) [1,2]. However, access-related complications, such as edema, bruising and cannulation pain, remain a relevant critical issue [3,4], especially in cases of delayed maturation or requiring surgery [5]. These physical problems can worsen over time, especially in long-term dialysis patients, and negatively affect satisfaction despite good patency [6,7].

Randomized controlled trials and meta-analysis have traditionally focused on clinical outcomes such as thrombosis, patency and infections [8]. Increasing attention has been paid to PROs, now recognized as critical indicators of quality in the management of vascular access [9]. Our findings reinforce this perspective: symptoms such as bruising and edema were associated with lower satisfaction, reflecting both physical and emotional impact.

In line with previous research [10,11], local complications were associated with impairment of daily activities, sleep disturbances and reduced mobility. Sikora et al. [12] confirmed that access-related symptoms can negatively affect quality of life and body image, especially if recurrent. In addition, our analysis shows that a longer duration of access use correlates with reduced satisfaction—probably due to accumulated complications or a sense of “fatigue” over time—a trend already reported in cohort studies [13,14]. In addition, the perception of hygiene of the access site emerged as a critical factor for satisfaction. This subjective perception may stem from fears of contamination, aesthetic discomfort, or distrust of daily care practices [15]. Although objective indicators such as bloodstream infections remain central [18], more studies recognize that the perception of cleanliness is equally important for patients [19,20].

Our data therefore confirm that satisfaction does not depend solely on objective clinical parameters but is also strongly influenced by subjective factors such as perception of hygiene, pain and esthetic impact. In this context, Yuo et al. developed the Arteriovenous Access Cosmesis Scale (AVACS), which assesses the esthetic and psychological impact of access, showing how scarring, skin changes and overall appearance can affect patient satisfaction and future choices [21]. A qualitative systematic review conducted by Casey et al. also showed that patients perceive access not only as a life-saving device, but also as a source of vulnerability, disfigurement and social stigma [22].

Our study also supports the importance of patient-centered approaches in vascular access management. Personalization of access choice—considering age, comorbidities, and quality of life—is consistent with modern nephrology guidelines [23,24]. These approaches encourage shared decision-making and periodic re-evaluation not only of functional aspects but also of the patient’s subjective experience.

The importance of individual preferences emerges clearly even in high-risk clinical settings. Mehandru et al. reported three cases of pregnant women who refused the creation of a fistula or graft, choosing to continue dialysis with a central venous catheter for reasons related to cannulation pain and aesthetic impact. Two of the three pregnancies ended positively, underlining how personal choices can be decisive and must be considered an integral part of therapeutic planning [25].

The role of the nurse remains crucial in shaping the patient experience, not only through cannulation technique and hygiene management, but also through communication and education [26]. The integration of PROs into nursing documentation can make it possible to identify early signs of dissatisfaction and to activate timely interventions. In addition, our data support the integration of multidisciplinary pathways that put clinical success and patient well-being on the same level [27]. Recent evidence also suggests that innovative interventions, such as the use of virtual reality during cannulation, can improve the experience by reducing procedural discomfort and increasing satisfaction [28].

Recent studies have shown that HRQoL is not determined so much by the type of access itself, but by the level of perceived satisfaction. Sridharan et al. found that while fistula was associated with greater satisfaction than catheter or graft, HRQoL did not vary between different types of access; it was satisfaction that explained much of the variance in quality of life [29]. A confirmation comes from the meta-analysis by Ahrabi et al., which documented how fistula is associated with greater global satisfaction than catheter, especially in social aspects and complications, while recognizing that in specific domains such as pain and bleeding some patients consider the catheter less burdensome [30].

Similar results also emerge in non-Western contexts. Asanova et al., in a study conducted in Kazakhstan, validated the KDQOL-SF™ and showed that the main predictors of quality of life were satisfaction with care, economic well-being, and positive dialysis experiences, while comorbidities and financial difficulties were the most influential negative factors [31]. In addition, Sass et al. highlighted how patients, caregivers and professionals attach great importance to comfort, privacy, costs and organizational flexibility, aspects often overlooked but central to overall satisfaction [32].

Our work is also part of the debate on organizational models and future prospects. The center in Tassin, France, represents a concrete example of how a global and multidisciplinary approach, centered on the patient and on the optimization of access, can translate into better clinical outcomes, with mortality halved compared to the United States [33]. At the same time, the most recent technological innovations—from endovascular fistulas to bioengineered grafts—show that even the most advanced research today recognizes the centrality of patient preferences and experience [34]. To support this effort, newly developed PROs specific to vascular access—such as the VASQoL scale—may help clinicians more accurately assess and monitor satisfaction over time [35,36].

Our study systematically documents how patient satisfaction with vascular access derives from the interaction between clinical parameters and subjective factors. Most of the literature research focused on clinical outcomes, while our study shows that patient perception is a crucial determinant of quality of life and adherence to treatment. This makes it essential to integrate PROs into care pathways, so that the success of vascular access is defined not only by its technical functionality, but also by its impact on the patient’s daily life.

An important consideration is the absence of objective clinical parameters in our analysis. Unlike studies that incorporate comorbidities, laboratory results, or dialysis adequacy measures, our investigation focused solely on PROs using the SF-VAQ. This aspect may be perceived as a limitation, but it reflects the novelty of our approach: systematically quantifying patients’ subjective satisfaction with vascular access, an aspect often underexplored in the nephrology literature. Our findings also provide complementary insights that extend beyond traditional clinical outcomes.

### 4.1. Limitations

This study has several limitations. First, its cross-sectional design only provides a snapshot of patient satisfaction at a single time point, limiting causal inferences regarding changes over time. The multicentric design and large sample size are key strengths of this study, enhancing the generalizability of our findings to broader dialysis populations. Additionally, the use of a validated tool (SF-VAQ) and rigorous statistical modeling strengthens the reliability of the results.

Second, there is a potential selection bias, as patients who were more satisfied with their vascular access may have been more likely to participate. Third, satisfaction with vascular access is a subjective measure and may be influenced by factors unrelated to clinical outcomes, such as individual expectations, psychological status, or dialysis staff-patient interactions. Finally, while we adjusted for relevant confounders, residual confounding cannot be entirely ruled out.

Another limitation is that we did not include clinical or laboratory data on causes of chronic kidney disease, history of previous vascular access, transplant records or measures of adequacy (e.g., Kt/V). This choice was intentional, as the study aimed to evaluate the satisfaction only through PROs. However, the absence of these objective parameters can reduce the comparability with other cohorts of patients and introduce other biases. Future studies can integrate PROs with clinical and laboratory variables to provide a complete evaluation of vascular access in hemodialysis.

### 4.2. Future Research Directions

While previous studies have primarily focused on clinical endpoints such as patency, infection risk, and technical failure, our research highlights the interaction between objective complications (e.g., edema, bruising) and patient-reported experience. It underscores the need for a more patient-centered approach to vascular access management.

The observed decline in perception with increasing vascular access duration highlights the importance of future studies exploring how patient perceptions and access-related complications evolve over time. Further research should assess whether strategies such as periodic reevaluation of access, structured hygiene education, and proactive complication management can mitigate declining perception scores over prolonged access use. Additional variables, such as elevated serum phosphorus or patient comorbidities, have also been associated with AVF dysfunction and may contribute to dissatisfaction in the long term. To our knowledge, this is one of the few multicenter studies that correlate PROs and access-related complications in Italian dialysis centers.

However, qualitative research based on patient interviews and focus groups could provide insights into the psychosocial dimensions of vascular access satisfaction. The knowledge of anxiety, body image concerns, and treatment fatigue contributes to dissatisfaction and can guide interventions to improve adherence and overall well-being.

Another potential research area involves the exploration of different types of vascular access impact on patient satisfaction. Since AVFs are generally superior in terms of clinical outcomes, long-term patency, and patient acceptability should be examined with different age groups, comorbidity levels, and dialysis vintage categories. To personalized vascular access choices, it could be conducting comparative studies assessing patient preferences for AVFs, AVGs, and CVCs.

## 5. Conclusions

This study demonstrated that local complications, perceived hygiene, and vascular access duration are significant determinants of patient satisfaction in hemodialysis. The integration of PROs into the daily management of vascular access can improve the proactive prevention of complications, personalized care, and patient education. Future multicenter studies are needed to confirm these findings, which can contribute to the formulation of patient-centered vascular access policies. The success of vascular access should be defined not only by technical patency but also by its impact on patients’ daily lives and quality of care.

## Figures and Tables

**Table 1:** Demographic and clinical characteristics of the study population (N = 235). Continuous variables are expressed as means (standard deviation), medians, and ranges. Categorical variables are presented as absolute frequencies and percentages.

Variable	Categories/Summary	Mean (SD)/%	Median	Range
Age (years)	-	68.27 (12.55)	70	20–91
Dialysis duration (years)	-	6.05 (5.70)	4	1–36
Number of children	-	1.78 (1.17)	2	0–11
Gender	Male	143 (60.85%)	–	–
	Female	91 (38.72%)	–	–
Marital status	Married	179 (76.17%)	–	–
	Single/Cohabiting/Divorced	10.21%/9.79%/3.83%	–	–
Children	Yes/No	86.38%/13.62%	–	–
Residence	Alone/With parents	15.74%/12.77%	–	–
	With spouse/With spouse and children/Nursing home	18.72%/40.85%/11.91%	–	–
Education level	None/Elementary	0.43%/4.26%	–	–
	Middle School/High School	37.87%/38.30%	–	–
	University/Specialization	14.47%/4.68%	–	–
Employment	Yes/No	12.34%/87.66%	–	–
Vascular access type	CVC/Native AVF/Prosthetic AVF	31.06%/61.28%/7.66%	–	–
Comorbidities	None/One/Two/> Two	48.09%/32.34%/12.34%/7.23%	–	–
Caregiver availability	Yes/No	24.26%/75.74%	–	–

**Table 2:** Results of the Short Form–Vascular Access Questionnaire (SF-VAQ) domains.

Item (SF-VAQ)	Mean ± SD
Q1. Overall satisfaction	6.28 ± 1.06
Q2. Pain	2.32 ± 1.81
Q3. Bleeding	2.25 ± 1.72
Q4. Edema	2.24 ± 1.78
Q5. Bruising	2.28 ± 1.84
Q6. ADL limitations	2.37 ± 1.74
Q7. Appearance	2.20 ± 1.59
Q8. Rest/Sleep	2.13 ± 1.55
Q9. Hygiene perception	2.17 ± 1.67
Q10. Problems	2.28 ± 1.67
Q11. Care	2.23 ± 1.73
Q12. Hospitalization	2.11 ± 1.68
Q13. Access duration	2.06 ± 1.54

**Table 3:** Correlation between demographic, clinical, and satisfaction-related variables. Pearson’s and Spearman’s coefficients are shown with corresponding *p*-values and 95% confidence intervals. Negative correlations indicate that higher symptom severity or longer vascular access duration were associated with lower satisfaction.

**Variables**	**Pearson’s R**	**Spearman’s Rho**	***p*** **-Value**	**95% CI**
Age vs. Dialysis duration	0.129	0.241	<0.05	[0.02–0.24]
Age vs. Number of children	0.352	0.384	<0.05	[0.23–0.46]
Satisfaction vs. Pain	−0.176	−0.204	<0.05	[−0.31–−0.02]
Satisfaction vs. Bleeding	−0.145	−0.168	<0.05	[−0.28–−0.01]
Satisfaction vs. Edema	−0.292	−0.315	<0.01	[−0.50–−0.08]
Satisfaction vs. Bruising	−0.236	−0.258	<0.05	[−0.44–−0.03]
Satisfaction vs. Hygiene perception	−0.183	−0.205	<0.01	[−0.29–−0.07]
Satisfaction vs. Vascular access duration	−0.324	−0.347	<0.01	[−0.55–−0.09]

**Table 4:** Predictors of patient satisfaction—Multiple linear regression. Negative β values indicate inverse associations between predictors and satisfaction scores.

Variable	Coefficient (β)	Std. Error	t-Value	*p*-Value	95% CI
Age	−0.0013	0.0049	−0.26	0.797	[−0.01–0.008]
Dialysis duration	−0.0047	0.0088	−0.54	0.592	[−0.02–0.01]
Number of children	−0.052	0.0460	−1.13	0.259	[−0.14–0.04]
Pain	0.029	0.0465	0.63	0.531	[−0.06–0.12]
Edema	−0.292	0.1047	−2.79	0.006	[−0.50–−0.08]
Bruising	−0.237	0.1032	−2.30	0.023	[−0.44–−0.03]
Hygiene perception	−0.184	0.0541	−3.39	0.001	[−0.29–−0.08]
Vascular access duration	−0.324	0.1165	−2.78	0.006	[−0.55–−0.09]

**Table 5:** Logistic regression model for high satisfaction. Odds ratios (OR) < 1 indicate reduced likelihood of high satisfaction.

Variable	Odds Ratio	Std. Error	z-Value	*p*-Value	95% CI
Hygiene perception	0.563	0.1046	−3.09	0.002	[0.39–0.81]
Vascular access duration	0.312	0.099	−3.67	<0.001	[0.17–0.58]
Number of children	0.651	0.128	−2.18	0.029	[0.44–0.96]

## Data Availability

The datasets generated and analyzed during the current study are available from the corresponding author upon reasonable request due to privacy and confidentiality restrictions.

## Supplementary Materials

The following supporting information can be downloaded at: https://www.mdpi.com/article/10.3390/clinpract15110203/s1. Supplementary Table S1: STROBE Statement—Checklist of items that should be included in reports of cross-sectional studies.
