# Supplementary material for: Determinants of Patient Satisfaction with Vascular Access in Hemodialysis: Insights from a Multicenter Study in Italy
Source: Clin Pract. 2025 Oct 31;15(11):203. doi: 10.3390/clinpract15110203 (PMC12650811; doi:10.3390/clinpract15110203)
Supplement: Supplementary file 1 [file clinpract-15-00203-s001.zip › clinpract-3863669-supplementary.pdf]

clinics and practice

MDPI

Table S1 - STROBE Statement—Checklist of items that should be included in reports of cross-sectional studies

Item	Checklist Item	Manuscript Location
**Title and abstract**	Indicate the study's design with a commonly used term in the title or the abstract.	Title, Abstract
**Introduction: Background/rationale**	Explain the scientific background and rationale for the investigation being reported.	Introduction
**Introduction: Objectives**	State specific objectives, including any prespecified hypotheses.	Introduction
**Methods: Study design**	Present key elements of study design early in the paper.	Methods
**Methods: Setting**	Describe the setting, locations, and relevant dates, including periods of recruitment, exposure, follow-up, and data collection.	Methods
**Methods: Participants**	Give the eligibility criteria, and the sources and methods of selection of participants.	Methods
**Methods: Variables**	Clearly define all outcomes, exposures, predictors, potential confounders, and effect modifiers.	Methods
**Methods: Data sources/measurement**	Give sources of data and details of methods of assessment (measurement).	Methods
**Methods: Bias**	Describe any efforts to address potential sources of bias.	Methods
**Methods: Study size**	Explain how the study size was arrived at.	Methods
**Methods: Quantitative variables**	Explain how quantitative variables were handled in the analyses.	Methods
**Methods: Statistical methods**	Describe all statistical methods, including those used to control for confounding.	Methods
**Results: Participants**	Report numbers of individuals at each stage of study.	Results
**Results: Descriptive data**	Give characteristics of study participants (e.g., demographic, clinical, social).	Results
**Results: Outcome data**	Report numbers of outcome events or summary measures.	Results
**Results: Main results**	Give unadjusted estimates and, if applicable, adjusted estimates and their precision.	Results
**Results: Other analyses**	Report other analyses performed, such as subgroup analyses and sensitivity analyses.	Results
**Discussion: Key results**	Summarize key results with reference to study objectives.	Discussion
**Discussion: Limitations**	Discuss limitations of the study, taking into account potential sources of bias.	Discussion
**Discussion: Interpretation**	Give a cautious overall interpretation of results.	Discussion
**Discussion: Generalizability**	Discuss the generalizability (external validity) of the study results.	Discussion
**Other information: Funding**	Give the source of funding and the role of the funders.	Funding Statement
